# Supplementary material for: The Potential Environmental Impact of PAHs on Soil and Water Resources in Air Deposited Coal Refuse Sites in Niangziguan Karst Catchment, Northern China
Source: Int J Environ Res Public Health. 2019 Apr 16;16(8):1368. doi: 10.3390/ijerph16081368 (PMC6518011; doi:10.3390/ijerph16081368)
Supplement: Supplementary file 1 [file ijerph-16-01368-s001.pdf]

Supplementary Material: The Potential Environmental Impact of PAHs on Soil and Water Resources in Air Deposited Coal Refuse Sites in Niangziguan Karst Catchment, Northern China

Chengcheng Li, Xin Zhang, Xubo Gao, Shihua Qi and Yanxin Wang

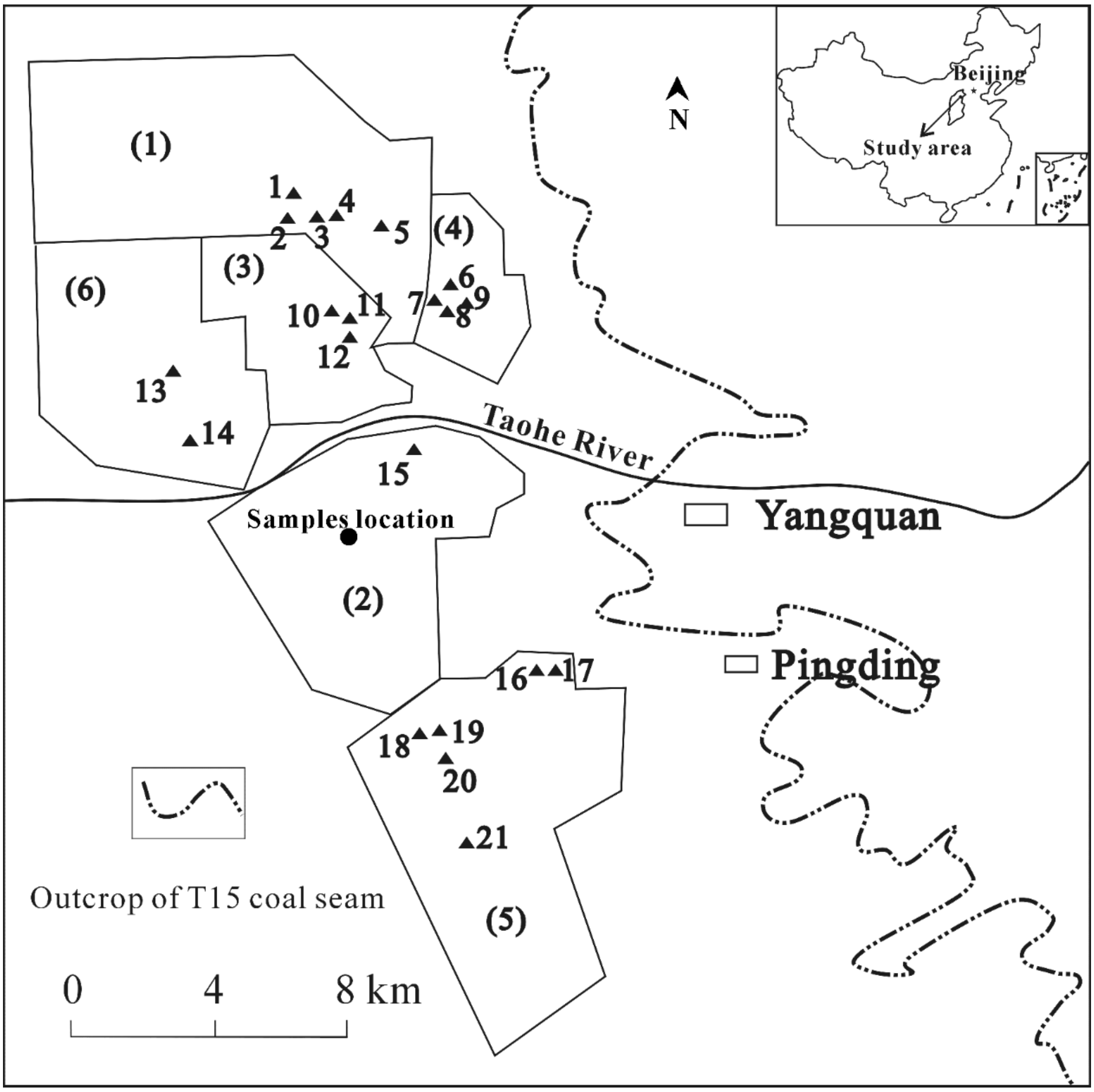

Figure S1. Location of major coal spoils piles in the study area. (1)–(6) stand for No. 1–5 coal mines and Xinjing coal mines; ▲ Coal spoils piles.

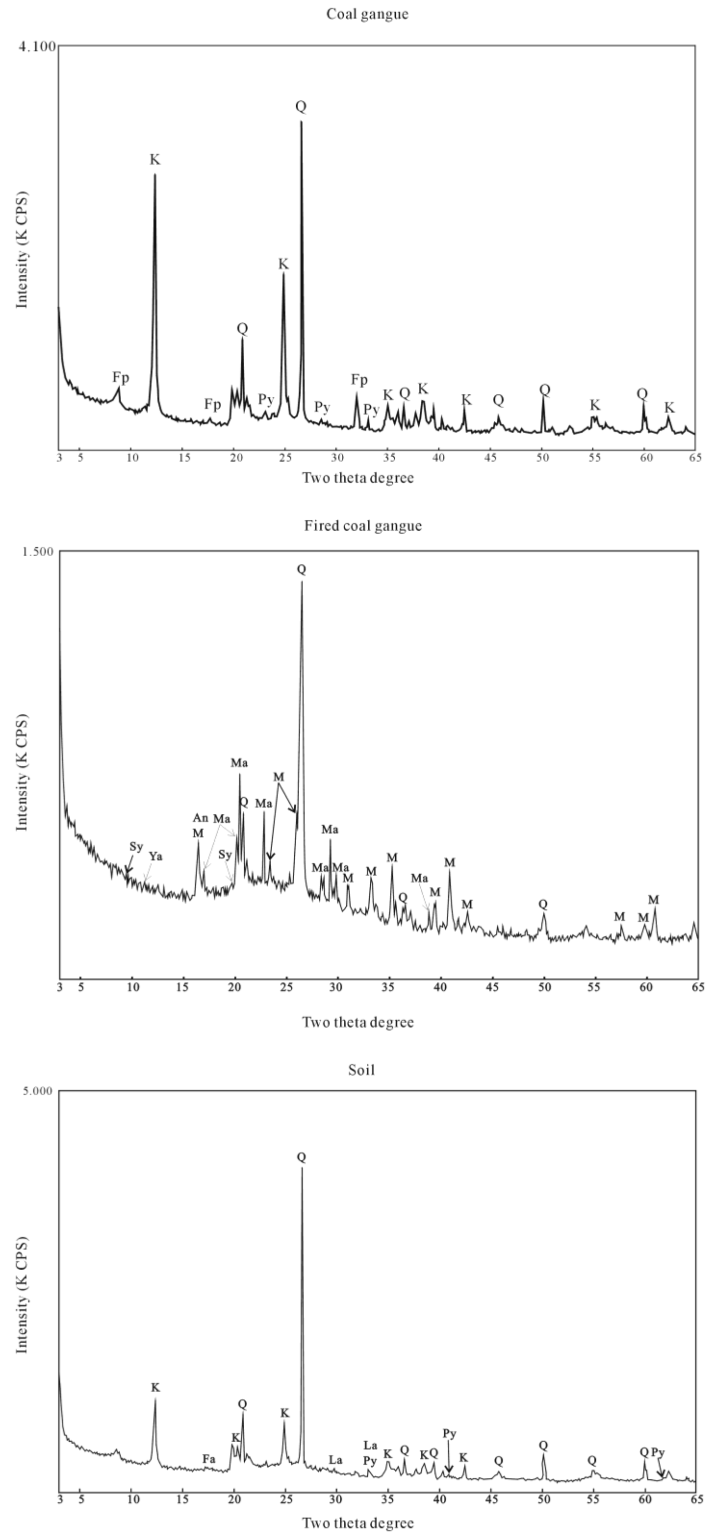

Figure S2. XRD patterns for coal gangue, fired coal gangue and contaminated soil. Q: quartz (SiO_2); K: kaolinite ($Al_2Si_2O_5(OH)_4$); M: mullite ($AlSi_2O_{13}$); Ma: mascagnine ($(NH_4)_2SO_4$); Py: pyrite (FeS_2); Fp: fluorphlogopite ($KMg_3(AlSi_3O_{10})F_2$); Sy: syngenite ($K_2Ca(SO_4)_2•H_2O$); Ya: Yavapaiite ($KFe(SO_4)_2$); Fa: ferroactinolite ($Ca_2Fe_5Si_8O_{22}(OH)_2$); La: lazurite ($Na_4Al_3Si_3O_{12}S$); An, anhydrite ($CaSO_4$).

Table S1. PAHs content release from coal spoils samples in heating/burning test under different temperature (ng/g, dry weight).

ID	T (°C)	Nap	Acy	Ace	Flu	Phe	Ant	Fla	Pyr	BaA	Chr	BbF	BkF	BaP	IP	DaA	BghiP	∑PAHs
H200	200	389.2	0.61	4.26	5.19	20.87	2.94	6.48	6.50	1.83	4.79	2.73	1.20	4.66	4.84	N.D.	1.03	457.1
H400	400	523.7	0.50	2.98	3.85	13.52	1.50	4.47	5.06	1.45	2.65	1.86	1.78	3.46	3.81	N.D.	1.55	572.1
H600	600	400.2	2.35	3.81	4.71	18.52	2.37	6.27	6.24	1.99	5.03	2.04	2.12	3.50	4.88	N.D.	4.26	468.3
H800	800	538.0	0.63	4.36	5.18	20.82	3.01	6.58	7.33	1.81	4.29	0.79	2.45	3.61	3.96	0.42	2.74	606.0
H1000	1000	198.5	0.89	3.82	5.02	19.27	2.03	8.04	9.21	2.55	4.98	3.12	2.43	3.52	3.95	N.D.	2.36	269.7
Av.1	-	409.9	1	3.85	4.79	18.6	2.37	6.37	6.87	1.93	4.35	2.11	2	3.75	4.29	0.42	2.39	474.6
STDEV1	-	136.5	0.77	0.54	0.56	3.01	0.63	1.27	1.54	0.40	0.99	0.90	0.52	0.51	0.53	N.D.	1.24	131.44
B200	200	343.5	0.57	3.83	4.96	18.45	1.43	5.95	6.11	1.71	3.35	1.94	1.24	2.02	2.52	N.D.	1.36	399.0
B400	400	149.0	3.19	4.53	5.84	21.95	2.39	7.48	6.82	1.92	3.60	0.14	1.69	1.39	0.00	N.D.	1.46	211.5
B600	600	36.06	1.01	4.51	5.91	24.12	2.43	8.15	8.32	0.70	0.07	0.59	0.09	0.62	1.32	N.D.	1.93	95.84
B800	800	46.31	1.82	3.62	4.57	21.68	2.01	4.67	5.42	1.92	2.22	0.42	0.74	2.05	1.36	N.D.	0.93	99.70
B1000	1000	159.4	0.19	1.62	1.76	7.50	6.64	2.67	2.93	0.81	1.80	0.37	1.05	2.29	0.89	N.D.	0.75	190.6
Av.2	-	146.9	1.36	3.62	4.61	18.74	2.98	5.78	5.92	1.41	2.21	0.69	0.96	1.67	1.22	N.D.	1.29	199.3
STDEV2	-	123.7	1.19	1.19	1.69	6.60	2.09	2.20	1.99	0.61	1.41	0.72	0.60	0.68	0.91	N.D.	0.46	123.2

Note: T is the temperature of heating or burning; H200-H1000 and B200-B1000 is the label for the heating test at different temperature (200, 400, 600, 800 and 1000 °C), respectively; N.D. not detected; - not data. Av.1 is the average value of PAHs release from coal spoils in the heating test; STDEV1 is the standard deviation value of PAHs release from coal spoils in the heating test; Av.2 is the average value of PAHs release from coal spoils in the burning test; STDEV2 is the standard deviation value of PAHs release from coal spoils in the burning test; All these statistical data is significant at the 0.05 level.
